# Agro-Industrial Potential of Yam (*Dioscorea* spp.): From Biological and Chemical Characterization to Industrial Applications and Valorization

**DOI:** 10.3390/foods15183178

**Published:** 2026-09-08

**Authors:** Katherine Paternina, Fabio Alfieri, Lesbia Cristina Julio-Gonzalez, Alexis López-Padilla, Piedad Montero, Francisco Javier Moreno, Oswaldo Hernandez-Hernandez

**Affiliations:** 1 IDAA Research Group, Faculty of Engineering, University of Cartagena, Cartagena 130015, Colombia; kpaterninas@unicartagena.edu.co (K.P.); ljuliog2@unicartagena.edu.co (L.C.J.-G.); pmonteroc@unicartagena.edu.co (P.M.); 2 Food Additives and Flavourings (FAF) Team, Food Ingredients and Packaging (FIP) Unit, European Food Safety Authority (EFSA), 43126 Parma, Italy; fabio.alfieri@efsa.europa.eu; 3 Institute of Food Science Research (CIAL, CSIC-UAM), 28049 Madrid, Spain; 4 Food Engineering Department, University of Cartagena, Cartagena 130015, Colombia; alopezp2@unicartagena.edu.co

**Keywords:** *Dioscorea*, yam, starch characterization, bioactive compounds, food security, agroindustrial valorization, postharvest technology

## Abstract

Yam (*Dioscorea* spp.) stands as a critical crop in tropical and subtropical regions, contributing significantly to food security, rural livelihoods, and cultural food systems. Beyond its traditional role as a subsistence crop, yam represents a biologically complex and agro-industrially strategic resource, characterized by substantial species diversity, nutritional richness, and functional versatility. This review provides an integrated synthesis of current knowledge on *Dioscorea* spp., with particular emphasis on *D. alata*, *D. rotundata*, and *D. cayenensis*. It examines their origin and domestication, global and regional production systems, agronomic requirements, morphological and genetic diversity, and chemical composition. Special attention is given to the nutritional profile of yam tubers, including starch, dietary fiber, proteins, vitamins, and minerals, as well as to their diverse array of bioactive compounds such as steroidal saponins, diosgenin, polyphenols, flavonoids, tannins, alkaloids, and dioscorin that underpin both functional and health-related properties. The review also addresses antinutritional factors and highlights the critical role of processing in mitigating potential toxicological effects while preserving or enhancing nutritional and bioactive value. Furthermore, emerging industrial applications of yam flour, starch, and phytochemicals are discussed, alongside current challenges and research opportunities for crop valorization, particularly in the Colombian context. Overall, this review highlights the potential of yam as a multifunctional crop and emphasizes the need for interdisciplinary strategies to advance its sustainable production, processing, and utilization in future agri-food systems.

## 1. Introduction

Yam (*Dioscorea* spp.) is a monocotyledonous plant of the genus *Dioscorea* (family *Dioscoreaceae*), encompassing 600–900 species, of which only 12 are considered edible [1]. Despite the limited taxonomic knowledge currently available for the family, more than 60 *Dioscorea* spp. are recognized as having economic importance worldwide. The most widely cultivated species include *D. alata*, *D. rotundata*, *D. cayenensis*, *D. trifida*, *D. villosa*, and *D. esculenta*, with *D. alata* being the preferred species for tuber production intended for human consumption [2,3]. In Colombia, *D. alata* and *D. rotundata* are regarded as the most important yam species in terms of cultivated area and market demand [4].

Beyond taxonomic diversity, *Dioscorea* spp. exhibit a highly variable nutritional profile, which is influenced by genetic background, environmental conditions, and methodological factors, including cultivar type, soil characteristics, agronomic practices, and analytical procedures [5]. In terms of proximate composition, *D. alata* (water yam), *D. rotundata* (white yam), and *D. cayenensis* (yellow yam) exhibit substantial variability, characterized by high moisture contents (often approaching 90%) and moderate protein levels. The dry matter of these species is dominated by carbohydrates, primarily starch with smaller contributions from sugars and dietary fiber, which collectively underpin the nutritional and technological properties of yam. Lipids and ash occur at lower proportions within the dry matter, contributing to the overall compositional profile and reflecting the characteristic macronutrient and mineral distribution of yam tubers [6,7,8].

In addition to their nutritional value, yam tubers are an important source of secondary metabolites, including phenolic compounds, flavonoids, tannins, saponins, alkaloids, and allantoin, which contribute to their functional and biological properties [6,9,10,11,12]. These compounds expand the relevance of yam beyond its role as staple food, positioning it as a raw material of interest for nutraceutical, pharmaceutical, and industrial applications.

Although several reviews have addressed the nutritional composition, phytochemistry, or health-promoting properties of yam, most have focused on specific topics or species. The present review aims to provide an integrated perspective encompassing agronomic, botanical, compositional, functional, and industrial aspects of yam. In addition, particular attention is given to the current status, challenges, and opportunities associated with yam production and valorization in the Colombian Caribbean. Particular emphasis is placed on *D. alata*, *D. rotundata*, and *D. cayenensis*, as these species represent the most important yam crops in Colombia in terms of cultivated area, commercial relevance, and contribution to regional food security and rural livelihoods. The review addresses their origin and domestication, production systems, morphological and genetic diversity, chemical and nutritional composition, including bioactive compounds and antinutritional factors, as well as their functional and biological properties and potential applications in food, pharmaceutical, and other industrial sectors.

Throughout this review, the names *D. alata*, *D. rotundata*, and *D. cayenensis* are used according to the terminology adopted in the original literature sources. The taxonomy of cultivated yam is complex, and *D. rotundata* and *D. cayenensis* are frequently regarded as members of the *D. cayenensis*–*D. rotundata* complex. For consistency and ease of comparison with the cited studies, the nomenclature used by the original authors has been retained throughout this review. Furthermore, species names are distinguished from cultivar names whenever possible. Thus, Criollo, Diamante, Espino, and Espino Mejorado are treated as cultivars commonly cultivated in Colombian production systems rather than as taxonomic entities.

## 2. Origin, Domestication, and Distribution of Yam (*Dioscorea* spp.)

The order Dioscoreales comprises a diverse group of monocotyledonous plants, among which *Dioscorea* represents the most species-rich genus within the family *Dioscoreaceae*, with more than 600 species currently accepted worldwide [13]. Phylogenetic evidence indicates that the evolutionary origin of the genus *Dioscorea* is located in the Laurasian Palearctic region, with diversification occurring between the Late Cretaceous and the Early Eocene. This early evolutionary phase was followed by multiple dispersal events toward the Southern Hemisphere during the Oligocene and Miocene, ultimately leading to the extensive pantropical distribution observed today [14]. These historical expansion processes account for the wide ecological amplitude and global distribution of *Dioscorea* spp.

The domestication of cultivated *Dioscorea* spp. represents a complex evolutionary process shaped by hybridization, polyploidy, and gene flow. These mechanisms have been facilitated by the predominantly dioecious reproductive system of the genus, which promotes obligate outcrossing and enhances genetic recombination [3]. At the global level, the most extensively domesticated and cultivated species are *D. rotundata*, *D. cayenensis*, and *D. alata*, which together account for the majority of worldwide yam production. Additional species of economic importance include *D. bulbifera*, *D. esculenta*, *D. opposita*, *D. japonica*, *D. nummularia*, *D. pentaphylla*, *D. transversa*, *D. trifida*, and *D. dumetorum* [2]. It should be noted that the taxonomic delimitation of *D. rotundata* and *D. cayenensis* remains debated, and both taxa are frequently treated jointly as the *D. cayenensis*–*D. rotundata* complex in germplasm, breeding, and agronomic studies. Nevertheless, because many nutritional, compositional, and food science studies continue to report them separately, both names are retained throughout this review when referring to the original literature sources.

In West Africa, genomic analyses have demonstrated that *D. rotundata* has a hybrid origin, resulting from genetic admixture between the wild progenitor species *D. abyssinica*, adapted to savanna ecosystems, and *D. praehensilis*, which is native to humid forest environments [3,15]. During the domestication process, *D. rotundata* was subjected to selection on genes associated with starch biosynthesis and storage, root and tuber development, and adaptation to higher light availability as cultivation progressively shifted from forested habitats to open agricultural fields.

The domestication of *D. cayenensis* is likewise linked to hybridization events, involving *D. rotundata*, already established as a cultivated species, and the wild species *D. burkilliana* [15]. In contrast, the domestication trajectory of *D. alata*, which originated in Asia, differs markedly from that of the African species. This species was primarily domesticated through vegetative propagation and processes of autopolyploidization rather than through interspecific hybridization. As a result, *D. alata* dispersed globally through prehistoric human migrations and later commercial trade routes, a process that has contributed to its relatively low levels of genetic diversity when compared with African yam species [3].

## 3. Morphological, Botanical, and Genetic Diversity of *Dioscorea* spp.

### 3.1. Key Morphological Characteristics

The species *D. alata*, *D. rotundata*, and *D. cayenensis* belong to the Enantiophyllum complex, a monophyletic group within Dioscorea characterized by twining vines with right-handed coiling and the formation of underground storage tubers [16,17]. Comparative morphological analyses indicate that leaves may be cordate or sagittate and display considerable variation in length, width, coloration, and venation patterns. Such variability has been widely documented, particularly in *D. alata* and *D. rotundata* [18,19,20].

**Figure 1:**
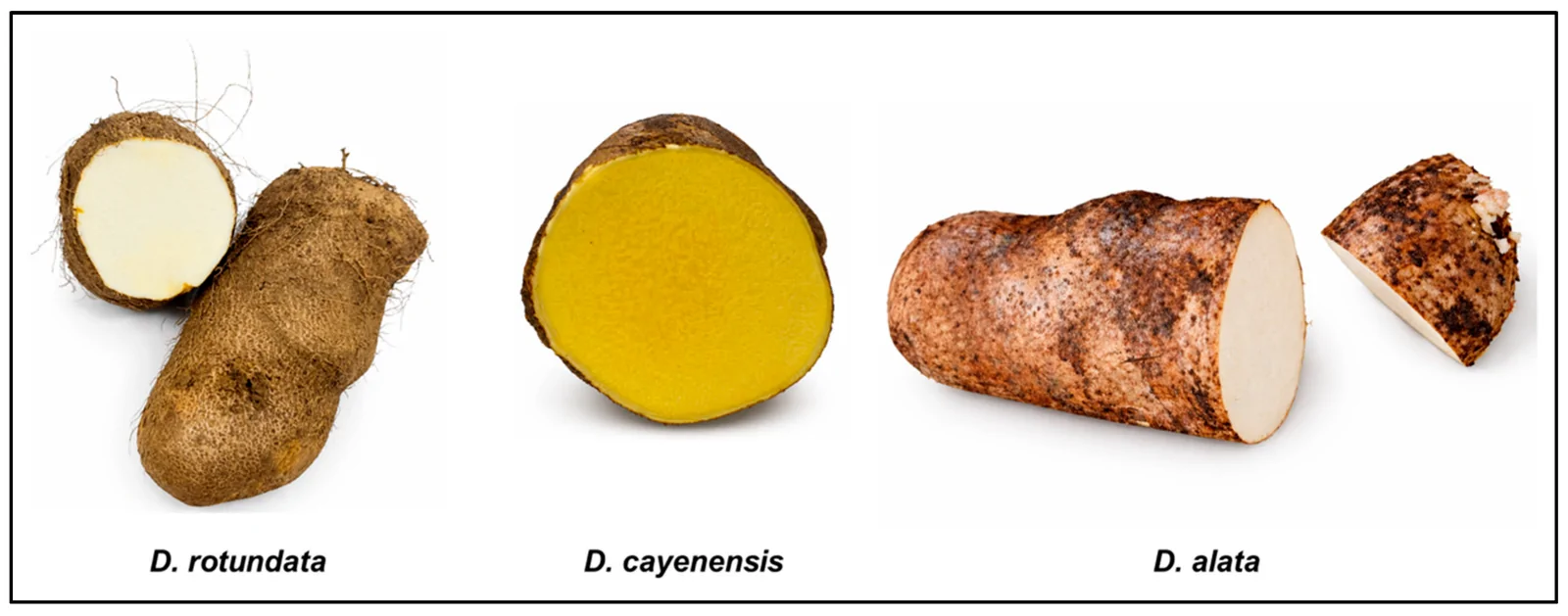
Representative tubers of yam species (*Dioscorea* spp.), illustrating general differences in external appearance and internal flesh among commonly cultivated varieties.

Stem morphology provides additional diagnostic traits for species and cultivar differentiation, including the presence or absence of membranous wings, varying degrees of spininess, differences in color and surface texture, and distinct growth architectures [21,22]. Tubers represent another key morphological component, differing in shape, ranging from cylindrical and oblong to round or oval, as well as in flesh color and texture. In *D. alata*, tuber flesh may be white, yellow, or purple, whereas white flesh predominates in *D. rotundata* (Figure 1) [18,19].

This morphological diversity, expressed in both vegetative organs and storage structures, supports species and genotype differentiation and constitutes a fundamental criterion for the evaluation, selection, and conservation of yam germplasm. A comparative summary of the main morphological and productive traits of three *Dioscorea* spp. cultivated in the Colombian Caribbean is presented in Table 1.

### 3.2. Genetic Resources and Conservation

Genetic diversity in cultivated and wild *Dioscorea* spp. underpins the sustainability and advancement of yam breeding programs. In *D. alata*, studies of genetic variability have revealed high levels of heterozygosity and complex population structure, resulting from successive domestication events and clonal propagation practices that restrict natural recombination [26].

Ex situ germplasm banks play a central role in conserving this diversity, as they enable the preservation of genetic materials that would otherwise be exposed to erosion due to varietal replacement, changes in production systems, or increasing phytosanitary pressures, risks that are particularly pronounced in tropical clonal crops [27]. Conservation strategies have increasingly incorporated in vitro techniques, which facilitate the maintenance of pathogen-free accessions while reducing the risks associated with exclusive field-based conservation. Micropropagation and microtuberization are now widely recognized as effective tools for preserving yam genetic resources and generating vegetative planting material with adequate sanitary quality [28]. In addition, cryopreservation of meristems and shoot apices has emerged as a long-term conservation alternative for vegetatively propagated species, allowing the safeguarding of genetic lines without the risk of degeneration or accidental loss [29].

In Colombia, conservation efforts remain limited when compared with those implemented in Africa and Asia. Institutions such as AGROSAVIA (the Colombian Agricultural Research Corporation) maintain *Dioscorea* germplasm collections under both field and in vitro conditions, which has enabled the preservation of regional varieties and introduced materials with agronomic potential [30]. However, several assessments highlight ongoing risks of genetic erosion driven by the replacement of traditional varieties, the lack of comprehensive molecular characterization, and the presence of diseases that compromise the sanitary quality of conserved materials. Moreover, most accessions have not been systematically evaluated for pathogen resistance or tuber quality attributes [30].

Local germplasm collections also face phytosanitary challenges, as tuber-propagated material can accumulate pathogens over successive cycles, reducing plant vigor and negatively affecting field productivity [31]. Consequently, the adoption of biotechnological approaches aimed at sanitation and rapid propagation represents a critical need for strengthening the conservation of yam genetic resources in Colombia.

### 3.3. Genetic Improvement and Applied Biotechnology

The availability and effective conservation of germplasm constitute the cornerstone of genetic improvement programs, providing the raw material for the development of new cultivars adapted to diverse production environments. At the global level, yam breeding is constrained by several biological characteristics intrinsic to *Dioscorea* spp., including dioecy, irregular flowering behavior, long growth cycles, and complex ploidy levels. These factors complicate the implementation of controlled crosses and limit genetic gain per breeding cycle [2].

To address these challenges, molecular tools such as simple sequence repeat (SSR) markers, single-nucleotide polymorphisms (SNPs), and quantitative trait loci (QTL) mapping have been increasingly integrated into yam improvement programs. These approaches facilitate the identification of genomic regions associated with traits of agronomic interest, including disease resistance and tuber quality attributes [2]. In parallel, gene-editing technologies such as Clustered Regularly Interspaced Short Palindromic Repeats/CRISPR-associated protein (CRISPR/Cas) have been proposed as emerging alternatives for introducing targeted genetic variation related to stress tolerance and nutritional quality [32].

In Colombia, genetic improvement of *Dioscorea* spp. has historically been limited, largely due to reduced investment in the crop and continued reliance on local varieties of *D. alata*, *D. rotundata*, and regionally adapted landraces from the Caribbean. Limited availability of high-quality planting material represents one of the principal constraints to breeding and crop expansion. Farmers are often required to allocate nearly one third of their harvested tubers for use as seed, which frequently exhibits physiological deterioration and phytosanitary problems that reduce productivity and increase vulnerability to pathogens [31].

National assessments emphasize that yam cultivation is strategic for food security and rural livelihoods and that there is substantial potential to broaden the genetic base used in improvement programs through the incorporation of introduced accessions and wild relatives, combined with the application of available molecular and biotechnological tools [30]. Nevertheless, the absence of systematic genetic characterization and the limited supply of high-quality planting material remain the principal barriers to the development and dissemination of improved yam varieties in Colombia.

## 4. Chemical Composition of Yam

Yam tubers are characterized by a chemical composition dominated by carbohydrates, primarily starch, together with smaller but nutritionally relevant amounts of proteins, fats, vitamins (especially vitamin C), and minerals such as phosphorus, potassium, calcium, magnesium, iron, and sodium [6,8,33,34].

Although more than 600 *Dioscorea* spp. have been described worldwide, only a limited number are widely consumed. Among these, *D. alata*, *D. rotundata*, and *D. cayenensis* exhibit the greatest commercial relevance and nutritional importance [35,36]. The nutritional composition of these species is influenced by genetic, environmental, and methodological factors, including cultivar, soil type, agronomic practices, and analytical procedures [5]. Differences in starch, fat, and ash contents have also been linked to variation in enzymatic activity during starch biosynthesis [7].

### 4.1. Carbohydrates

Carbohydrates, primarily starch, together with dietary fiber and minor amounts of sugars, dominate the proximate composition of yam (Table 2) and are central to its nutritional and technological value [37]. Carbohydrates not only provide metabolic energy but also determine the tuber’s functionality in food processing.

#### 4.1.1. Starch

Starch constitutes 60–85% of yam dry matter, with its physicochemical properties largely dictating the quality of yam-based foods. Interestingly, the starch content and functionality of different *Dioscorea* spp. are affected by environmental and agronomic conditions, extraction methods, and tuber maturity [33,37].

Structurally, starch consists of two polymers: amylose and amylopectin. Amylose is predominantly linear, composed of α-D-glucose units joined by α (1 → 4) bonds, conferring gelling and thickening capacity [38,39]. Amylopectin, by contrast, is highly branched through α (1 → 6) linkages, imparting viscosity and structural complexity. Yam starch typically contains approximately 20–30% amylose and 70–80% amylopectin, depending on species [38,40,41,42].

The technological potential of yam starch lies in these molecular features. The amylose/amylopectin ratio strongly influences thermal, pasting, and textural properties [35,43,44]. Nevertheless, in comparison with starches from potato, cassava, and sweet potato, yam starches remain under-studied, which has limited their industrial exploitation and the recognition of yam as a sustainable crop [42,45,46,47].

During gelatinization, the semicrystalline structure of starch granules is disrupted as they absorb water and swell, forming gels through the release and rearrangement of amylose and amylopectin molecules [48,49]. Upon cooling, these chains reassociate, increasing crystallinity and firmness in a process known as retrogradation [50,51]. Low-amylose starches gelatinize more readily and produce softer gels, whereas high-amylose starches tend to retrograde more extensively and generate greater quantities of resistant starch [44,52]. This characteristic is nutritionally relevant, as resistant starch modulates the glycemic index of foods.

Resistant starch behaves physiologically as a component of dietary fiber [53,54]. It is increasingly incorporated into food formulations aimed at reducing caloric density and glycemic response [53,55]. Moreover, resistant starch exerts prebiotic effects, selectively stimulating the growth of beneficial gut microorganisms, such as *Lactobacillus* and *Bifidobacterium* spp. [52,56]. Consequently, resistant starch has been associated with beneficial effects on gut microbiota composition and metabolic health. However, the magnitude of these effects depends on dose, food matrix, and study population. Functionally, resistant starch exhibits a neutral flavor, white color, fine particle size, and favorable rheological properties, attributes that facilitate its application as a dietary-fiber and prebiotic ingredient in health-promoting foods.

#### 4.1.2. Dietary Fiber

Dietary fiber is essential for digestive health, intestinal microbiota modulation, and reduced colon cancer risk [35,37,57]. Regular dietary fiber intake increases fecal bulk, enhances intestinal transit, and helps prevent constipation. In the colon, fiber fractions, particularly fermentable components, serve as substrates for beneficial microbial populations, thereby supporting microbial diversity and gut health.

Compared with other tropical root and tuber crops such as potato, cassava, and sweet potato, yam generally exhibits a higher dietary fiber content, reinforcing its nutritional relevance among staple foods [37]. However, it is important to distinguish between dietary fiber, which reflects physiological functionality, and crude fiber, which is an analytical fraction determined by conventional proximate analysis and represents only a portion of total dietary fiber. Reported crude fiber values for yam tubers typically range from approximately 0.4 to 1.9% (fresh weight, FW), depending on species and cultivar [7,8]. These values largely reflect insoluble structural components and tend to underestimate total dietary fiber content. Nevertheless, certain yam varieties exhibit crude fiber levels comparable to those of whole-wheat flour, highlighting their potential contribution to fiber intake.

From a compositional perspective, yam fiber comprises both insoluble components, including cellulose, hemicellulose, lignin, and pectic substances, and soluble fractions, such as mucilages and non-digestible oligosaccharides. The relative proportions of these fractions vary across species and cultivars, influencing both the physiological effects of yam consumption and the functional behavior of yam-derived ingredients in food systems.

#### 4.1.3. Soluble Sugars

In addition to starch and dietary fiber, yam tubers contain modest but nutritionally relevant amounts of soluble sugars, which influence sweetness, postharvest physiology, and sensory properties of yam-based foods [35,37]. Although starch predominates, soluble sugars, including reducing and non-reducing forms, constitute a small and variable fraction of total carbohydrates. Their concentration may increase during storage as starch is enzymatically hydrolyzed [6,8].

In *D. rotundata*, and to a lesser extent in *D. alata* and *D. cayenensis*, total sugar content is generally low to moderate, representing only a few percent of dry matter [6,8]. During postharvest storage, reducing sugar levels often rise as dormancy ends and sprouting begins, reflecting carbohydrate mobilization [8,35]. The magnitude of this increase depends on species, genotype, maturity, and storage conditions [37].

From a nutritional perspective, soluble sugars contribute to palatability but exert limited influence on the overall glycemic index of yam products, which is primarily determined by starch composition [37]. However, during thermal processing, these sugars participate in Maillard reactions, influencing color, flavor, and the formation of both desirable and undesirable browning products [35,57]. Excessive accumulation of reducing sugars prior to processing may therefore negatively affect product quality.

### 4.2. Proteins

Proteins fulfil crucial structural and regulatory roles, maintaining tissue integrity and governing metabolic processes [6,58]. Adequate intake contributes to a balanced diet and provides essential amino acids. Although protein levels in yam are modest compared with legumes or cereals, they are notable among root crops. Essential amino acids, such as phenylalanine and threonine, are present in appreciable amounts, while tryptophan and sulphur-containing amino acids (cysteine, methionine) are limiting [6]. Therefore, yam consumption should be complemented with other protein-rich foods [37].

Dioscorin, the predominant storage protein in yam, accounts for most of its soluble protein fraction. In addition to its nutritional role, dioscorin has been associated with various biological activities, which are discussed in detail in Section 5.

### 4.3. Lipids

*Dioscorea* spp. are characterized by very low lipid contents, a feature that contributes to their low energy density from fats and supports their classification as carbohydrate-dominant staple foods. Across major cultivated species, including *D. alata*, *D. rotundata*, and *D. cayenensis*, crude fat levels are consistently reported at trace to minimal concentrations, generally ranging from 0.03 to 0.27% on a FW basis [7,8]. These low values reflect the biological role of yam tubers as starch storage organs and the limited accumulation of neutral lipids in their parenchymatous tissues.

Although lipids are quantitatively minor, they may still play functional roles at the cellular level, particularly as constituents of membrane structures and lipid-associated bioactive compounds. From a technological perspective, the limited lipid fraction contributes to the good storage stability of yam flours and starches, as low-fat levels reduce susceptibility to oxidative rancidity during processing and storage.

### 4.4. Ash and Mineral Composition

The ash content of yam, which provides an indirect estimate of total mineral matter, is generally modest but shows notable interspecific and varietal variability (Table 3). Reported ash values for fresh yam tubers typically range from 0.6 to 2.2% on a FW basis, depending on species, cultivar, soil characteristics, and agronomic conditions [6,8]. Higher ash contents are commonly associated with increased accumulation of nutritionally relevant macrominerals, reflecting the mineral-uptake capacity of different yam species and their growing environments.

Beyond ash as a global indicator, yam tubers are recognized as valuable sources of both macro- and micro-elements, particularly potassium, magnesium, calcium, and phosphorus [52]. Potassium is typically the predominant mineral in yam tubers and plays an important role in physiological functions such as electrolyte balance, renal regulation, and blood-pressure control [59,60,61]. Magnesium, although present at lower concentrations than potassium, contributes significantly to metaboli functions, acting as a cofactor in numerous enzymatic reactions and supporting neuromuscular and energy metabolism [62]. Calcium, which may be present at substantial levels relative to other divalent minerals, can interact with soluble dietary fiber and other polysaccharides, forming complexes that influence the textural and rheological properties of yam-based foods [7]. Phosphorus in yam tubers may occur partly in association with starch as phosphate monoesters, introducing negative charges that enhance starch swelling power and paste viscosity, with implications for both nutritional quality and technological functionality. Together, these minerals contribute to the overall nutritional value of yam and reinforce its role as a mineral-containing staple food that complements its high carbohydrate content. Overall, the variability observed in both ash and mineral composition emphasizes the importance of species selection, soil fertility management, and post-harvest handling practices in determining the mineral quality of yam tubers and yam-derived products. This variability also highlights opportunities for targeted agronomic and breeding strategies aimed at enhancing the micronutrient contribution of yam within sustainable food systems.

### 4.5. Vitamins

Vitamin composition in yam varies according to species, soil and climatic conditions, and post-harvest handling. Generally, yam is rich in water-soluble vitamins, particularly ascorbic acid (vitamin C) and B-complex vitamins, such as thiamine (vitamin B_1_) and pyridoxine (vitamin B_6_) [37,57,58]. Vitamin C levels are usually higher than in cassava and comparable to those in potato [37]. This vitamin is essential for collagen synthesis, iron absorption, immune defense, bone growth, and antioxidant protection. However, its concentration decreases with prolonged storage or exposure to heat and light [37,63,64]. The B-complex group contributes to energy metabolism and neurological functions and supports erythropoiesis [37]. Yellow-fleshed species, particularly *D. cayenensis*, also contain β-carotene (provitamin A), which supports vision and immune function [57,58,65]. In summary, yam may be considered a moderate source of vitamin C and B_6_, while yellow varieties additionally provide carotenoids. The preservation of these micronutrients depends on cool, dark storage and gentle cooking methods [37].

### 4.6. Antinutritional Factors of Yam

Raw yam tubers contain several antinutritional factors, including oxalates, phytates, tannins, saponins, and certain alkaloids [37,66]. These compounds have been associated with reduced protein and carbohydrate digestibility, impaired mineral absorption, potential adverse physiological effects, and bitter taste when tubers are consumed without adequate processing [35,67,68,69]. Collectively, their presence highlights the importance of appropriate processing and preparation prior to consumption, as they influence both the nutritional quality and safety of yam-based foods.

#### 4.6.1. Oxalates

Oxalates occur naturally in plant tissues but provide no known nutritional benefit. At elevated levels, they may cause tissue irritation and gastrointestinal discomfort, manifesting as burning sensations in the mouth, throat, or skin and, in severe cases, swelling [37,68,70]. Owing to their high chelating capacity, oxalates reduce the bioavailability of essential minerals, including iron, calcium, zinc, and magnesium.

Oxalate concentrations vary among yam species and depend on analytical methods and expression basis (Table 4). Additional studies conducted on wild and underutilized *Dioscorea* species have also reported substantial interspecific variation in oxalate content [71]. For the species considered in this review (*D. alata*, *D. rotundata*, and *D. cayenensis*), the literature sources summarized in Table 4 indicate relatively low oxalate contents, although substantial variability among studies has been reported. Such variation may reflect differences in genotype, environmental conditions, sample preparation, analytical methodology, and expression basis. However, thermal processing, particularly boiling, substantially reduces oxalate content, with reported reductions ranging from about 30% up to 40–50%, depending on cultivar, tuber size, and boiling time, primarily due to enhanced solubility and leaching of oxalates at elevated temperatures [66,70]. Consequently, when appropriate processing methods are applied, yam consumption does not pose a toxicological risk associated with oxalate intake [68].

#### 4.6.2. Phytates

Phytates act as antinutritional factors by chelating essential minerals in the gastrointestinal tract, thereby reducing their bioavailability [68,74]. In the cultivated species considered in this review, phytate concentrations reported in the selected studies were generally low (Table 4), although considerable variation has been reported across the *Dioscorea* literature [74]. Such variability may arise from species- and genotype-dependent effects as well as from differences in analytical methodology and reporting basis. Therefore, direct comparisons among studies should be interpreted with caution. Nevertheless, thermal processing methods, such as boiling, substantially reduce phytate levels. Moreover, moderate dietary phytate intake has been associated with potential health benefits, including reduced risks of cardiovascular disease and type 2 diabetes [68].

#### 4.6.3. Tannins

Tannins are polyphenolic compounds widely distributed in plant tissues, including yam [8,75]. In *Dioscorea* spp., tannins generally occur at moderate levels (Table 4) and contribute primarily to sensory attributes, notably bitterness and astringency, which influence consumer perception and acceptance of yam-based products [8]. Astringency arises from the ability of tannins to form complexes with salivary and dietary proteins, leading to protein precipitation and the characteristic dry or puckering mouthfeel associated with tannin-rich foods [75].

At elevated concentrations, tannins may affect nutritional quality through interactions with proteins, digestive enzymes, and minerals. These interactions include reduced protein utilization, inhibition of digestive enzymes such as trypsin, amylase, and lipase, and interference with iron absorption, potentially posing dietary challenges in populations where tannin-rich foods constitute dietary staples [8,37,57,68,75].

Thermal processing like cooking markedly reduces tannin concentrations, largely through leaching and structural modification. Because tannin levels in properly processed yam remain well below intakes associated with adverse effects, yam poses no meaningful risk to human health with respect to tannin intake [68].

As with other phytochemicals, the balance between beneficial and antinutritional effects of tannins depends on concentration, chemical structure, dietary context, and processing history. Thermal treatments reduce tannin levels and astringency, thereby improving palatability and minimizing potential adverse nutritional effects. Potential biological and functional properties of tannins are discussed in Section 5.5.

#### 4.6.4. Alkaloids

Alkaloids constitute a diverse class of nitrogen-containing secondary metabolites derived primarily from amino acids [9,76]. In *Dioscorea* spp., alkaloid concentrations vary widely among species and genotypes, with wild yam species generally exhibiting higher levels than cultivated varieties [9,58] (Table 4). Alkaloids may exert antinutritional effects through their activity on the nervous system and their potential to disrupt gastrointestinal cell membranes [6,8,37]. In combination with saponins, alkaloids also contribute to the characteristic bitterness of yam tubers [73]. Potential biological activities of yam alkaloids are discussed in Section 5.7.

#### 4.6.5. Saponins

Saponins are naturally occurring glycosidic secondary metabolites widely distributed in plant tissues, including yam, where they are particularly abundant in tubers and, to a lesser extent, in peels and leaves [6,37]. Structurally, saponins consist of a hydrophobic aglycone (sapogenin), typically steroidal in *Dioscorea* spp., linked to one or more hydrophilic sugar moieties. This amphiphilic nature underlies their antinutritional effects [35,77].

From a nutritional perspective, saponins are considered antinutritional factors primarily because of their ability to impart bitterness and astringency, which can reduce palatability and consumer acceptance of yam-based foods [65,73]. The bitter taste associated with certain yam cultivars has been directly linked to saponin concentration, often acting synergistically with alkaloids [73]. In addition, saponins can interact with dietary proteins and cell membrane sterols, potentially affecting nutrient absorption and gastrointestinal integrity when consumed in high amounts [6,37].

Furthermore, saponins may interfere with nutrient absorption when consumed at high levels [72,78]. Quantitative analyses indicate that saponin concentrations in edible yam species are generally low to moderate (Table 4) and remain well below levels associated with acute toxicity [73]. Nevertheless, saponins may contribute to the characteristic bitterness of yam tubers and can cause mild gastrointestinal discomfort when tubers are consumed raw or inadequately processed [35,68]. The distinction between the bioactive and antinutritional roles of saponins is not defined by a single concentration threshold. Rather, it depends on their structural characteristics, dietary exposure, food matrix, and processing conditions. In edible yam species, thermal processing substantially reduces saponin concentrations while generally retaining levels compatible with safe consumption and potential biological activity. The occurrence and biological relevance of steroidal saponins are discussed further in Section 5.1.

In addition to these compounds, enzyme inhibitors such as trypsin inhibitors and α-amylase inhibitors have been reported in certain *Dioscorea* spp., particularly in wild or under-utilized yams, where they may impair protein and starch digestibility; however, their levels are generally low in cultivated edible varieties and are substantially reduced by thermal processing [6,37,71].

Overall, the concentration of these antinutritional factors in edible yam species remains low and well within safe dietary limits [37,68]. Nevertheless, certain wild species, notably *D. hispida*, contain higher levels of toxic alkaloids, such as the toxic alkaloid dioscorine, highlighting the necessity for correct species identification and adequate heat processing prior to consumption [35].

#### 4.6.6. Processing Strategies for the Mitigation of Antinutritional Factors

Appropriate processing techniques are essential for ensuring the safety of yam-based foods while preserving their nutritional value. Conventional methods such as boiling, blanching, and soaking are highly effective in reducing antinutritional factors, particularly oxalates, tannins, alkaloids, and saponins, largely due to their water solubility and thermal sensitivity. Among these methods, boiling has consistently been shown to produce the greatest reductions, often exceeding 90%, resulting in substantial improvements in edibility and safety [35,68,69,78].

Comparative studies indicate that boiling is more effective than other thermal treatments across yam species and cultivars in reducing oxalates, phytates, tannins, and alkaloids [68,78]. For example, oxalate reductions of 40–50% have been reported in *D. alata* tubers following boiling, whereas steaming and baking result in more modest decreases of approximately 20–25% and 12–15%, respectively [79]. Boiling has also been shown to reduce saponin levels by more than 60% in *D. alata*, primarily through leaching into cooking water and thermal degradation [73]. Comparable reductions have been observed following blanching and soaking, although their effectiveness depends on factors such as processing duration, tuber size, and species [65,68].

In addition to saponins, boiling markedly lowers alkaloids and tannins, thereby eliminating most potential toxicological risks and improving palatability [35]. Soaking treatments also demonstrate beneficial effects: Sahoo et al. [69] reported significant reductions in antinutrient levels after four hours of soaking, although prolonged soaking may lead to losses of water-soluble nutrients [80,81].

Overall, simple and widely adopted household practices, including peeling, boiling, blanching, soaking, and sun- or oven-drying, represent practical, low-cost strategies for reducing antinutritional load and enhancing the sensory and nutritional quality of yam products [35,68]. These findings underline that potential toxicological risks associated with yam consumption are primarily linked to inadequate preparation rather than to the inherent composition of edible *Dioscorea* spp. When standard domestic or industrial processing methods are applied, yam is a safe, wholesome, and nutritionally valuable food.

## 5. Bioactive Compounds of Yam

Following the discussion of antinutritional factors and their mitigation through processing, it is important to recognize that many of the same secondary metabolites responsible for bitterness or reduced nutrient bioavailability also confer significant health-promoting properties when present at appropriate concentrations and consumed in properly processed forms. This dual functional–antinutritional behavior represents a defining feature of yam phytochemistry. Whether these compounds act predominantly as bioactive or antinutritional constituents depends not only on their concentration but also on their chemical structure, processing history, dietary exposure, and interaction with the food matrix. Representative examples of bioactive compounds identified in yam are summarized in Table 5.

It should be noted that much of the available evidence regarding the biological activities of yam-derived compounds originates from studies using isolated compounds, enriched extracts, cell-culture systems, or animal models. Therefore, these findings should not be interpreted as direct evidence of health benefits resulting from the consumption of yam as a food. Human clinical studies remain limited, and further research is required to establish the relevance of these observations under normal dietary conditions.

Although particular emphasis is placed on *D. alata*, *D. rotundata*, and *D. cayenensis*, information from other *Dioscorea* species is incorporated where appropriate to provide a broader perspective on the occurrence and biological relevance of bioactive compounds within the genus. These constituents have been associated with antioxidant, anti-inflammatory, antimicrobial, and other biological effects in experimental models, contributing to the growing interest in yam-derived compounds for food, nutraceutical, and pharmaceutical applications [91]. Collectively, this phytochemical diversity underscores the importance of *Dioscorea* spp. not only as staple food crops but also as valuable sources of nutraceutical compounds.

### 5.1. Steroid Saponins

While saponins may negatively affect palatability or nutrient utilization at high levels or in insufficiently processed tubers, steroidal saponins are among the most biologically significant bioactive metabolites in yam. Their characteristic structure, comprising a steroidal aglycone linked to one or more sugar moieties, underpins their diverse biological and pharmacological activities [37,85]. The occurrence and diversity of steroidal saponins across *Dioscorea* spp. have been extensively documented through compositional and analytical studies [6,77,84,85].

More than 50 steroidal saponins have been identified in *Dioscorea* spp. and are primarily classified into furostanol, spirostanol, and pregnane-type skeletons. The relative abundance and composition of steroidal saponins differ markedly among *Dioscorea* species. Species such as *D. zingiberensis* are recognized as particularly rich sources of steroidal saponins and diosgenin precursors, which explains their importance in pharmaceutical applications. In contrast, cultivated food species generally contain lower concentrations but still contribute significantly to the overall bioactive profile of yam. Notably, 21 compounds have been isolated from methanolic extracts of *D. villosa* and *D. cayenensis*, including protodioscin, methyl protodioscin, and dioscin [37,85]. Advanced analytical techniques, such as two-dimensional NMR spectroscopy, have further revealed substantial structural complexity within yam saponin profiles [77].

This structural diversity is directly associated with a broad spectrum of biological activities. Steroidal saponins have demonstrated anti-inflammatory, hypolipidemic, antifungal, antioxidant, and cardioprotective effects in experimental studies [52,85]. Mechanistically, these effects include modulation of proinflammatory cytokine secretion, improvement of endothelial function, and protection against oxidative stress [37,85]. In addition, emerging evidence suggests a role for steroidal saponins in gut health through interactions with intestinal microbiota and enhancement of gastrointestinal protection [52,83].

From a functional and industrial perspective, steroidal saponins are of particular interest. For example, dioscin exhibits anti-inflammatory, anticancer, and antimicrobial properties [77,78], whereas diosgenin-rich saponins display antioxidant activity and have been associated with potential roles in the prevention of metabolic and cardiovascular disorders; moreover, they are widely exploited as precursors for steroid hormone synthesis [92,93]. Because diosgenin has received particular attention due to its pharmaceutical relevance and industrial importance, it is discussed separately below.

### 5.2. Diosgenin

Diosgenin is a naturally occurring steroidal sapogenin (the sugar-free aglycone of steroidal saponins such as dioscin and protodioscin) and is primarily extracted from the roots of various *Dioscorea* spp. [76]. Its chemical structure is closely related to that of endogenous steroids such as cholesterol, progesterone, and estrogen, which underlies its significant pharmacological relevance, particularly in hormone replacement therapy and the industrial semi-synthesis, via the classic Marker degradation, of steroid medications such as norethindrone and cortisone [82,94]. Diosgenin has been investigated for multiple biological activities, including anti-inflammatory, antimicrobial, and cholesterol-lowering effects, primarily in experimental models [76,95].

Structurally, diosgenin contains a hydroxyl group at the third carbon position, contributing to its saponaceous behavior and influencing its solubility characteristics [95]. In plants, the biosynthesis of diosgenin proceeds through a complex pathway in which cholesterol serves as a key precursor and is catalyzed by specific cytochrome P450 enzymes. Although its solubility in aqueous media is very low, consistent with its high lipophilicity (calculated log P ≈ 5.7), diosgenin readily dissolves in organic solvents such as ethanol and dimethylformamide, facilitating its extraction and purification [95].

Diosgenin is predominantly sourced from approximately 15 species within the *Dioscorea* genus, with major production occurring in China and Mexico, which together account for roughly 67% of the global supply [37,76]. Its estimated market value of approximately USD 500 million reflects its importance within both the pharmaceutical and nutraceutical industries [37].

Available evidence suggests substantial interspecific differences in diosgenin content. Species traditionally exploited for pharmaceutical purposes, such as *D. zingiberensis* and *D. deltoidea*, generally contain considerably higher levels than most cultivated food yams. Consequently, species selection remains a critical factor when considering industrial diosgenin production. These differences are likely associated with species-specific genetic backgrounds and domestication histories, as cultivated food yams have generally been selected for agronomic performance and edibility rather than for the accumulation of specialized metabolites.

The steroidal saponins of *Dioscorea* share a common C27 spirostane or furostane skeleton, and their interconversion helps explain why yam preparation matters so much. Furostanol glycosides such as protodioscin carry a sugar at both C-3 and C-26; removal of the C-26 glucose, whether by processing or by the tuber’s own β-glucosidases, closes the F-ring to give the spirostanol saponin dioscin. Subsequent hydrolysis of the C-3 sugar chain then releases the aglycone diosgenin [83]. Because each step depends on cooking, storage, and enzyme activity, the diosgenin ultimately recoverable from a tuber can vary widely with how it is handled. A better understanding of these enzymatic transformations may support the development of more sustainable strategies for diosgenin recovery and utilization.

### 5.3. Polyphenols

Polyphenols comprise a diverse class of metabolites characterized by their phenolic structures, which play key physiological roles in plants, including pigmentation, defense against environmental stressors, and interactions with other macromolecules. In yam, polyphenols include flavonoids, phenolic acids, lignans, and stilbenes, all of which contribute to the tuber’s recognized health-promoting potential. Consistent evidence indicates that yam extracts exhibit antioxidant, anti-inflammatory, and antimicrobial activities, with total phenolic content strongly correlated with free-radical scavenging capacity [6,58,96].

Polyphenol profiles vary considerably among species, cultivars, and tissues. Peels frequently contain higher concentrations than edible flesh, while environmental conditions and processing practices can further influence phenolic composition and antioxidant capacity.

The physiological impact of these polyphenols is strongly influenced by their behavior within the food matrix and during digestion. Polyphenols interact with dietary polysaccharides and other macronutrients through covalent and non-covalent mechanisms, including hydrogen bonding and electrostatic interactions, which can modify their stability, release, and bioavailability. These interactions affect polyphenol metabolism in the gastrointestinal tract and help explain their roles in gut microbiota modulation, gastrointestinal protection, and cardiometabolic health [6,58]. Consequently, the functional properties of yam polyphenols depend not only on their intrinsic chemical structures but also on matrix-dependent transformations occurring during digestion [58]. The broader health implications of polyphenol intake include reported roles in blood-glucose regulation, weight management, and anticancer activity. Collectively, these attributes underscore the relevance of yam polyphenols in nutraceutical development and highlight their potential as key contributors to functional-food applications [6,58].

### 5.4. Flavonoids

Multiple yam species contain appreciable levels of flavonoids [37,78,86]. These compounds constitute a major group of polyphenolic constituents widely present in the human diet and are recognized for their strong antioxidant properties. Their chemical structure, characterized by multiple aromatic rings, underpins a wide range of biological activities. Such compounds play an important role in mitigating oxidative stress, which is implicated in chronic diseases such as cardiovascular disorders and cancer [86,97]. Flavonoids can directly scavenge free radicals and enhance endogenous antioxidant defenses, providing additive protective effects [86,97]. They may also indirectly modulate cellular antioxidant status by influencing signaling pathways involved in oxidative stress responses, with interactions involving reactive oxygen species among the most well-characterized mechanisms of action [97].

Differences in flavonoid composition among *Dioscorea* species may contribute to variation in antioxidant potential, color characteristics, and stability during processing, highlighting the importance of species-specific characterization.

Beyond antioxidant effects, flavonoids have demonstrated anti-inflammatory activity, gastrointestinal protection, modulation of gut microbiota composition, and anticancer effects through regulation of cell proliferation and apoptosis in cell and animal models. In yam, these metabolites significantly enhance the overall bioactive potential of the tubers, reinforcing their relevance as functional foods [78,86,97].

### 5.5. Tannins

Beyond their antinutritional role discussed in Section 4.6.3, tannins exhibit several biological activities. In particular, tannins possess antioxidant properties that contribute to cellular protection against oxidative stress, supporting their relevance as bioactive constituents in yam [76]. In addition, both traditional and experimental studies have reported potential therapeutic roles for tannins, including applications in the management of conditions such as leucorrhea and diarrhea [75].

The functional behavior of tannins is strongly influenced by their interactions with other biomolecules present in the food matrix. Polysaccharides, for example, can compete with salivary proteins for tannin binding, thereby reducing perceived astringency and modifying sensory response. Structural characteristics of tannins, including monosaccharide composition and linkage patterns, govern the formation of tannin–protein and tannin–polysaccharide complexes and ultimately shape their bioavailability and biological effects [76].

### 5.6. Dioscorin

Dioscorin is a major bioactive protein found in many yam species and serves both as the predominant storage protein and an important contributor to nutritional quality [37,98]. It accounts for a very large proportion of the soluble protein fraction in yam, representing approximately 85–90% of extractable proteins in several *Dioscorea* spp., and in some cases exceeding 90%, thereby making an important contribution to the protein quality of the tuber [35,52,89]. The relative abundance of dioscorin varies among *Dioscorea* spp. and genotypes, indicating that its nutritional contribution is strongly genotype-dependent [37,98].

It is worth distinguishing dioscorin from the similarly named dioscorine: the former is the abundant, largely benign tuber storage protein discussed here, whereas dioscorine is a toxic azaspirocyclic alkaloid found mainly in wild species such as *D. hispida*. The two are unrelated in both structure and function, and the resemblance of their names is a frequent source of confusion in the literature.

In addition to its nutritional role, dioscorin exhibits a wide range of biological activities, including antioxidant, immunomodulatory, estrogenic, trypsin-inhibitory, and carbonic anhydrase activities [57,89,90]. Additional functional properties, such as chitinase-like, insecticidal, acaricidal, lectin-like, and antiproliferative activities, have also been reported, highlighting its potential therapeutic and protective roles [99]. Moreover, dioscorin has been shown to enhance the bioactivity of other yam-derived compounds and to protect cells against oxidative stress, thereby contributing to physiological homeostasis and supporting its relevance in health-related applications, including hypertension management [89,90,98].

### 5.7. Alkaloids

Beyond their antinutritional properties described in Section 4.6.4, alkaloids present in yam have been linked to a range of health-related biological activities. Extracts of *D. alata* have demonstrated cytotoxic activity against human cancer cell lines, while *D. oppositifolia* has shown anti-ulcer activity in animal models [9,76]. Additional pharmacological properties include analgesic, antispasmodic, and antibacterial effects, underscoring the potential of yam alkaloids in drug discovery and therapeutic development [58,76].

Future research should prioritize controlled human studies to clarify alkaloid bioavailability, pharmacokinetics, and mechanisms of action, as well as their potential applications in treating serious diseases. Improved understanding of these compounds may support the development of novel therapeutic agents, particularly in the context of antimicrobial resistance and other emerging health challenges [58,76].

## 6. Growing Conditions and Environmental Requirements for Yam Cultivation

Yam can be cultivated under a wide range of edaphoclimatic conditions typical of tropical and subtropical regions, a characteristic that underpins its broad geographic distribution and agro-food relevance [6]. Among the environmental factors influencing yam growth, temperature is particularly critical. The crop is sensitive to temperatures below 20 °C and does not tolerate frost, while optimal growth generally occurs between 25 and 30 °C, a range that promotes vegetative development. However, excessively high temperatures may delay tuber initiation and development [100,101].

Water availability is another key determinant of yam productivity at the ecological level. Although yam plants can tolerate short periods of drought, sustained reductions in soil moisture negatively affect growth and yield. Yam cultivation is therefore associated with regions characterized by adequate and relatively stable rainfall regimes and is generally feasible at altitudes of up to approximately 1000 m [101,102]. In addition, photoperiodicity influences the physiological balance between vegetative growth and tuber formation, with longer day lengths favoring leaf development and shorter days promoting tuberization [102].

## 7. Agronomic Practices: Soil Preparation, Planting Systems, and Crop Management

Planting density in yam cultivation varies according to species, cultivar, and management system, typically ranging from 10,000 to 20,000 plants ha^−1^ [103,104]. Recent agronomic evaluations have demonstrated that the interaction between planting density and staking systems in *D. rotundata* significantly affects total tuber production and spatial efficiency, underscoring the importance of appropriate planting design as a yield-optimization strategy [104].

From a management perspective, water availability constitutes a critical agronomic consideration that shapes planting calendars and crop performance. Species such as *D. alata* and *D. rotundata* perform adequately under annual rainfall levels of 1000–1500 mm distributed over six to seven months, whereas *D. cayenensis*, which has a longer growth cycle, requires rainfall periods extending for at least nine months [101,102]. Photoperiodic responses also influence agronomic decision-making, as shorter day lengths favor tuberization while longer days promote vegetative growth [102].

Staking, crop rotation, and fertilization critically influence yam productivity. Optimal agronomic performance is generally achieved in loamy soils with high organic matter content, good drainage, and pH values close to neutrality [105,106]. Nutrient management studies indicate that nitrogen and potassium fertilization significantly enhance tuber production, whereas phosphorus application tends to have a limited effect on yield, highlighting the importance of targeted fertilization strategies [102,107]. These relationships have been documented across environmental gradients in West Africa, where management intensity strongly influences yield outcomes [107]. Cultivar selection and sanitary management are equally important, particularly given the high phenotypic variability observed in *Dioscorea* spp., which affects traits such as tuber development, drought tolerance, and overall productivity [108].

Among recent technological innovations adopted in Colombian yam production systems, the use of plant growth–promoting rhizobacteria (PGPR) has received increasing attention. Experimental trials with *D. rotundata* demonstrated that inoculation with *Azotobacter chroococcum* (DBC12) or *A. vinelandii* (DBC9), combined with 50% of the recommended nitrogen dose, produced the highest yields and increased the proportion of first-quality tubers. These findings indicate that PGPR application can replace up to 50% of conventional nitrogen fertilization without compromising yield or tuber quality, offering a promising alternative for smallholder systems with limited access to agricultural inputs [109].

## 8. Yield Determinants, Harvesting Practices, and Postharvest Handling

Yam yield and tuber quality are determined by the interaction among genotype, soil conditions, water availability, nutritional management, plant health, and the physiological quality of planting material. Physiological and genetic studies emphasize the importance of varietal selection and phenotypic traits associated with productivity, including tuber size, plant architecture, and biomass conversion efficiency [108]. In the Colombian Caribbean, a major constraint identified is the genetic deterioration of traditional planting material, coupled with limited access to high-quality seed, which increases susceptibility to diseases and reduces production stability [110].

Harvesting of yam is generally carried out between nine and ten months after planting, when foliage senescence indicates physiological maturity of the tubers. This harvest window is widely documented in traditional *Dioscorea* spp. production systems across the Caribbean and tropical America [111]. At maturity, tubers are highly susceptible to mechanical damage; therefore, harvesting is typically performed manually and with caution to minimize cuts and impacts that may facilitate infection by postharvest pathogens such as *Lasiodiplodia theobromae* (*syn. Botryodiplodia theobromae*), *Aspergillus flavus*, and *Penicillium* spp. [111].

Postharvest handling critically determines storage life and quality. Careful handling during transport, physical protection of tubers, and the implementation of hygienic practices are essential to reduce deterioration. Curing under warm and well-ventilated conditions promotes wound healing, while subsequent storage under controlled temperature and humidity reduces losses associated with molds, rots, and dehydration [112,113].

In many areas of the Colombian Caribbean, limited infrastructure for harvesting, handling, and storage exacerbates postharvest losses. These constraints are often intensified by phytosanitary problems, including anthracnose, whose incidence is closely associated with the technological level of local production systems. In this context, Martínez-Reina [114] demonstrated that training processes focused on early symptom recognition, pathogen identification, and risk factor awareness significantly improved farmers’ diagnostic capacity. Their work with local communities, including the active participation of women in agriculture, showed that strengthening these competencies enhances the perception of phytosanitary risks and supports more effective decision-making, reducing the underestimation of diseases such as anthracnose.

## 9. Global Patterns of Yam Cultivation, Production, and Socioeconomic Relevance

Yam serves as a staple food for millions across tropical and subtropical regions, holding major socioeconomic importance and ranks as the fourth most important tuber crop worldwide. Its significance is underpinned by its high energy contribution, wide range of culinary uses, and strategic role in food security and rural livelihoods, particularly in West Africa, the Caribbean, Asia, and Oceania [115,116]. At the global scale, yam production was estimated at approximately 73 million tons in 2018, with Africa accounting for 94–97% of total output. Nigeria is the world’s leading producer and exporter [115,116]. More recent data indicate that global production reached about 89 million metric tons in 2023, harvested from approximately 10.5 million hectares. Production remains highly concentrated in Africa, which contributes approximately 97% of the global total, with Nigeria producing 62.4 million metric tons, followed by Ghana (10.5 million), Côte d’Ivoire (7.5 million), and Benin (3.3 million metric tons) [117].

**Figure 2:**
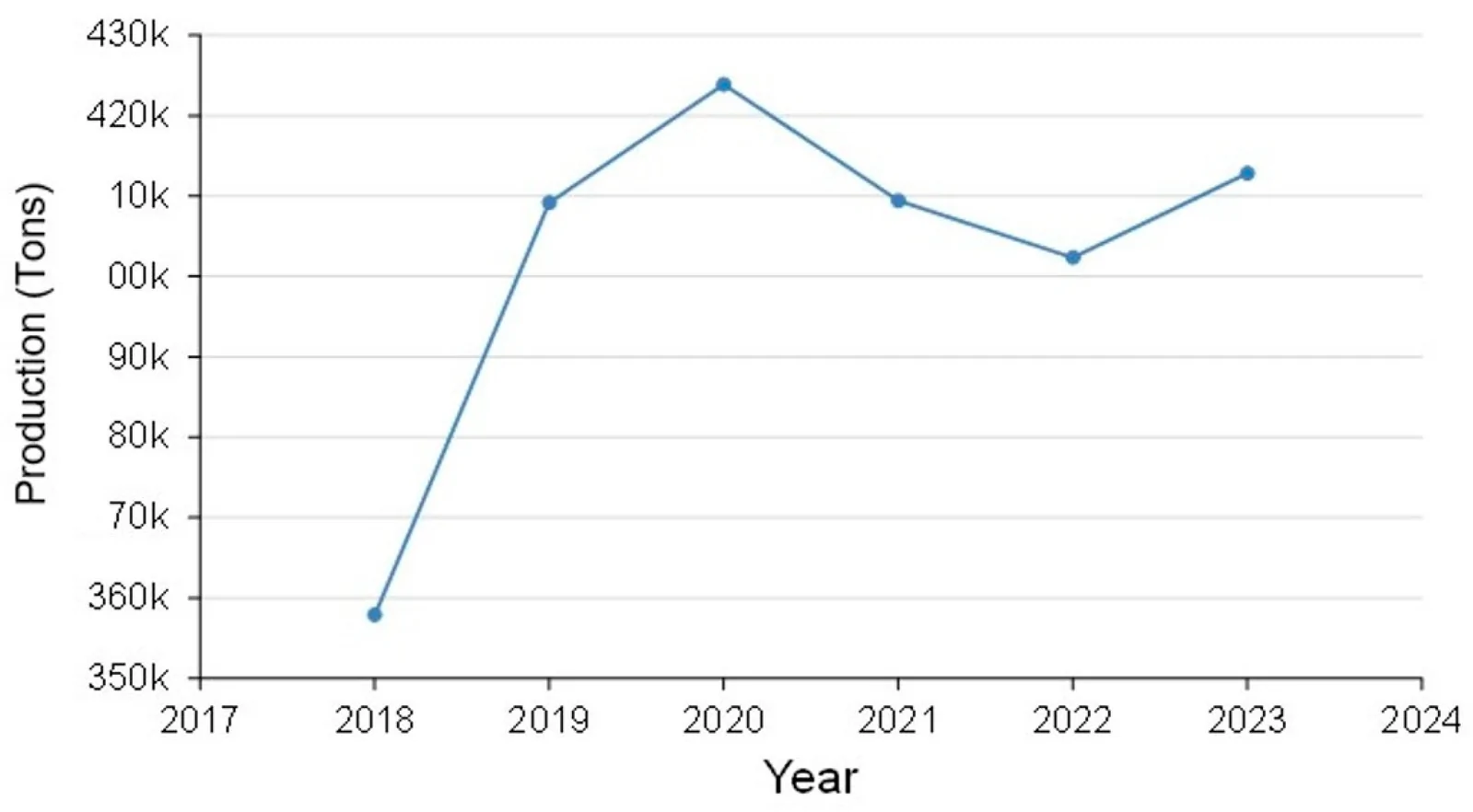
Trends in Colombian yam production from 2018 to 2023. Adapted from [117].

In Latin America, Colombia is the leading yam producer and ranked eighth worldwide in 2023 (ninth in 2018). National production increased from 357,902 metric tons harvested from 28,799 ha in 2018 to 412,819 metric tons harvested from 35,725 ha in 2023 (Figure 2) [117,118]. Cultivation is concentrated almost exclusively in the Caribbean region, which accounts for approximately 96% of the national cultivated area. The main producing departments, Córdoba, Bolívar, and Sucre, are characterized by small, family-based production systems in which yam represents a key source of income for small- and medium-scale farmers. Average production units are approximately 1.3 ha, reflecting the predominantly smallholder nature of the crop [119,120].

Production in the Colombian Caribbean is dominated by *D. alata* and *D. rotundata*, particularly the cultivars Criollo, Diamante, Espino, and Espino Mejorado, which constitute the main regional supply [118]. Cultivar-specific data indicate that mean cultivated areas are approximately 0.55 ha for Espino, 0.67 ha for Diamante, and less than 0.40 ha for Criollo [36,118]. The practice of cultivating multiple cultivars within the same production unit is common, particularly in Bolívar and Antioquia, whereas greater varietal diversity and more complex cultivar combinations are observed in Córdoba and Sucre, reflecting region-specific management strategies [118].

Among the cultivated species, *D. alata*, including locally adapted landraces, is especially valued for its adaptability and strong consumer preference [121]. Beyond its role as a staple food, yam exhibits considerable agro-industrial potential, providing carbohydrates, proteins, and minerals and serving as a raw material for starch extraction, bioproduct development, and compounds of pharmaceutical interest. The majority of production (87.3%) is directed to the market, while smaller proportions are retained for seed (12.4%) or used in barter (0.34%) [118].

## 10. Key Challenges, Constraints, and Development Opportunities for Yam Production in Colombia

Notwithstanding its importance for food security, income generation, and rural livelihoods, the yam production system in Colombia is characterized by a low level of technological development, widespread use of local planting material, reliance on traditional practices, and limited adoption of innovations related to soil management, pest and disease control, and harvest and postharvest improvement. These structural and technological constraints primarily affect on-farm productivity and production stability. In addition, the continued harvesting of several *Dioscorea* spp. from wild populations exerts pressure on fragile ecosystems, underscoring the need for targeted conservation strategies. These challenges have been consistently documented for the Caribbean region, where insufficient technical assistance and limited institutional support negatively affect productivity and profitability [10,118,122].

Anthracnose, a widespread fungal disease, represents one of the main phytosanitary constraints affecting yam cultivation in the Caribbean region, especially for the Criollo cultivar, whose susceptibility has led to a marked decline in its cultivation. This vulnerability limits its suitability for monoculture systems and has favored its replacement by more disease-tolerant materials [10,122].

Postharvest limitations and market access restrictions further exacerbate production challenges. Due to limited logistical capacity and infrastructure, marketing often relies on intermediaries, a common feature of smallholder systems that generates price variability and restricts producers’ access to higher-value markets [123].

Despite these constraints, yam production in Colombia presents clear development opportunities at the commercialization level, particularly in international fresh produce markets. Evidence of increasing global yam trade over the past decade highlights its export potential [10]. Colombia has consolidated its position among international suppliers of fresh yam, with commercial presence in markets such as the United States [124]. In addition, producers in the department of Sucre have identified the Miami (Miami, FL, USA) market as a strategic destination for export expansion [125].

The main characteristics, challenges, market opportunities, and research needs associated with yam production in the Colombian Caribbean are summarized in Box 1.

Box 1 **Key facts, challenges and opportunities for yam production in the Colombian Caribbean.**

Colombia is the main yam producer in Latin America with production increasing from approximately 315,000 tons in 2018 to more than 412,000 tons in 2023. Cultivation is concentrated in the Caribbean region and is mainly carried out by smallholder farmers, highlighting its importance for food security and rural livelihoods.

 

**Production characteristics:** Yam cultivation is based on traditional systems using locally adapted cultivars, mainly the species *Dioscorea alata* and *D. rotundata*. Production depends largely on vegetative propagation and is oriented toward commercialization as fresh tubers, with limited agroindustrial transformation.

 

**Major constraints:** Anthracnose is one of the main phytosanitary limitations, reducing yield and tuber quality. In addition, continuous use of vegetative planting material contributes to pathogen accumulation. Postharvest losses remain high due to inadequate handling, storage, and limited infrastructure.

 

**Market opportunities:** Yam has stable national demand and increasing international market potential. The development of value-added products such as flour, starch, and functional ingredients represents an important opportunity.

 

**Research priorities:** Key needs include the development of disease-resistant cultivars, production of pathogen-free planting material, improvement of postharvest technologies, and strengthening of processing and value-chain integration.

## 11. Industrial Applications of Selected Yam Species (*D. alata*, *D. rotundata*, *D. cayenensis*)

The industrial applications of the yam species *D. alata*, *D. rotundata*, and *D. cayenensis*, particularly within the food and pharmaceutical sectors, stress the significance of these tubers in global agriculture and health [37,58]. These species are extensively cultivated for their starchy tubers, which serve as vital sources of nutrition and energy for millions, particularly in regions such as Latin America and the Caribbean, West Africa, and Southeast Asia [126]. *D. alata* (water yam) is recognized for its culinary versatility, nutritional richness, and suitability for processing [59,127], while *D. rotundata* (white yam) is a dietary staple in Africa and Latin America. *D. cayenensis* (yellow yam) is valued for its traditional uses, including medicinal applications, and its technological potential, particularly in starch-based applications [37,126,128].

Each of these yam species contains characteristic bioactive compounds that support their growing relevance in industrial innovation, including antioxidant, anti-inflammatory, and antimicrobial properties. These attributes enhance their potential as functional and nutraceutical ingredients, contributing to increasing interest from the pharmaceutical industry, where yam-derived compounds are explored for incorporation into health supplements and drug formulations. For instance, starch isolated from yam can serve as a key raw material in the development of biodegradable materials and natural polymers for pharmaceutical uses. This is particularly evident for *D. cayenensis* starch, which has been evaluated for binder and disintegrant properties in tablet manufacturing, demonstrating its effectiveness as an excipient capable of improving hardness, disintegration time, and overall tablet performance [37,58]. Further advances in sustainable processing technologies, including enzyme-assisted approaches, may contribute to improving the recovery and functionality of yam-derived ingredients for industrial applications.

In the food industry, *D. alata* is commonly processed into flour, which functions as a thickener, stabilizer, and texturizer in various food formulations [59,129]. Although production has traditionally relied on artisanal methods, modern processing techniques are increasingly being implemented to improve efficiency and product quality [128,129]. Yam starch is also widely used in the food industry as a thickening and stabilizing agent in numerous products [58].

In contrast, *D. rotundata* has shown promise in non-food industrial applications, such as the manufacture of refractory and insulating materials. This potential is supported by its thermal resistance and compatibility with local clay minerals, particularly in Nigeria [130].

Reflecting the expanding industrial interest in yam-derived compounds, recent patents highlight diverse applications across the cosmetic, pharmaceutical, and food sectors (Table 6). These patents involve several *Dioscorea* spp., including *D. alata*, *D. oppositifolia*, and *D. zingiberensis*, all recognized for their high diosgenin content and other valuable bioactive constituents. Together, these developments reinforce the industrial relevance of yam as a renewable biological resource [37,58].

The patents summarized in Table 6 demonstrate that yam valorization is advancing beyond its traditional role as a staple food crop toward the development of high-value products for the cosmetic, pharmaceutical, nutraceutical, and functional food sectors. The diversity of patented applications, particularly those involving diosgenin-rich extracts and steroidal saponins, highlights the commercial relevance of yam-derived bioactive compounds and their potential for industrial innovation. Furthermore, patent activity serves as an indicator of increasing technological interest and market opportunities, suggesting that future yam valorization strategies may benefit from integrated approaches combining food processing, bioactive compound recovery, and product development. These trends are particularly relevant for producing countries such as Colombia, where the development of value-added yam-based products could contribute to economic diversification and strengthen the competitiveness of the yam value chain.

## 12. Enzyme-Assisted Strategies for Yam Valorization

Conventional yam processing still relies mainly on drying, milling, boiling, autoclaving and extrusion. These operation techniques are useful, but they mostly modify the tuber through heat, pressure or mechanical disruption, with limited control over the molecular changes that finally affect digestibility and functionality. Enzyme-assisted processing are more selective alternative. Amylases, pullulanase, cellulases, pectinases, proteases and glycosidases can be used under relatively mild conditions to target specific components of the yam matrix, allowing the modification of starch, the release of bioactive compounds or the recovery of fermentable sugars from residues. This is especially attractive for yam because its composition can vary considerably between species, cultivars and growing conditions. It also fits well with current green processing and circular bioeconomy approaches, since enzymes can reduce the need for severe chemical or thermal treatments.

One of the most developed applications is the production of starch with lower digestibility. Pullulanase hydrolyzes the α-(1 → 6) branching points of amylopectin, generating shorter linear chains that can reassociate during retrogradation and form more ordered structures. In purple yam (*D. alata*), pullulanase treatment combined with autoclaving increased amylose content from about 30% to 42% and raised relative crystallinity to 31% [136]. The resulting starch was more resistant to digestion by α-amylase and amyloglucosidase and showed a lower estimated glycemic index. Interestingly, the same resistant starch improved the tolerance of *Bifidobacterium adolescentis* under simulated gastric and bile conditions and increased short-chain fatty acid production [137], which suggest a possible prebiotic use. Enzymatic treatments can also interact with other operations. For example, extrusion combined with an enzymatically produced soy-protein hydrolysate increased starch crystallinity and reduced in vitro digestibility [138]. In another direction, amylase and glucoamylase can be used to hydrolyze yam starch into reducing sugars, and this process has been modelled using artificial neural networks to improve process control [139]. Similar principles are already applied industrially in resistant maltodextrins and enzyme-resistant starches, as reflected in patents such as CN100445301C [140], WO2006041851A1 [141]. and US20170335020A1 [142], although their conditions would need adaptation to yam starch.

Enzymes can also improve the recovery of compounds that remain trapped inside the tuber matrix. A clear example is diosgenin production from *D. zingiberensis*. Enzymatic saccharification removed around 98% of the starch, making steroidal saponins more accessible, and subsequent fermentation with *Trichoderma reesei* produced a diosgenin yield of 90.6%, around 42% higher than direct bioconversion of untreated tubers [143]. The process was reproduced at 5 L scale, which gives it some practical relevance. This saccharification followed by biotransformation is consistent with the furostanol → spirostanol → sapogenin pathway described in Section 5.2 and may reduce part of the acid and solvent demand associated with conventional diosgenin production. The same idea can be extended to polyphenols, flavonoids and polysaccharides, since cellulases and pectinases can partially break the cell wall and improve the accessibility of compounds present in tuber and peel tissues.

For Colombia, however, residue valorization may be one of the more realistic applications in the short term. Peels, damaged tubers, trimmings and solids remaining after extraction still contain starch and other carbohydrates that are normally underused. A recent Colombian techno-economic study applied ultrasound-assisted enzymatic hydrolysis to starch remaining after anthocyanin extraction and recovered fermentable sugars for a small biorefinery scheme [144]. A similar approach could be applied to yam residues generated in the Colombian Caribbean, converting part of this material into sugars, oligosaccharides or modified starches instead of treating it only as waste. The main advantage is not that enzymes will replace all conventional processing, but that they can be inserted where more selective transformation is needed.

## 13. Bottlenecks and Innovation Needs for Yam Valorization in Colombia

Beyond primary production constraints, yam valorization in Colombia remains limited by the predominance of fresh-tuber commercialization, low levels of agroindustrial transformation, and insufficient postharvest and processing infrastructure, despite the significant potential of yam-derived ingredients and products. Yam is commonly consumed boiled, fried, roasted, baked, or mashed; however, preparation methods vary by region and species. Cooking has been reported to decrease total phenolic and diosgenin contents, with little effect on allantoin. Steaming enhances the bioaccessibility of phenolic compounds (71.2%) and allantoin (79.1%), whereas high-pressure boiling promotes higher diosgenin retention (75.6%) [145]. These findings emphasize the need for research aimed at optimizing processing conditions to preserve bioactive and functional compounds, particularly in view of expanding industrial and health-related applications.

Postharvest and pre-processing operations represent additional bottlenecks for yam valorization. Manual peeling and handling may cause dermal irritation, while trimming or grading often induce enzymatic browning and quality deterioration. Addressing these issues requires the development of safer, standardized, and sustainable pre-processing technologies, such as edible coatings, vacuum packaging, and modified-atmosphere packaging, to extend shelf life and facilitate industrial utilization [52].

Yam flour represents an important value-added product. However, its broader utilization remains constrained by variability in physicochemical properties, processing conditions, and product standardization requirements. Future research should therefore focus on optimizing particle size and drying techniques to improve nutrient bioaccessibility while balancing product quality and production costs [52].

Yam starch constitutes another high-potential derivative for crop valorization, although its industrial use remains limited. Studies have shown that starch from *D. rotundata* improves viscosity and water retention when used as a stabilizer in fermented dairy products [10,146]. Nevertheless, native starches often present functional limitations due to granule heterogeneity [147]. These constraints could be overcome through physical, chemical, or enzymatic modification approaches, enabling the production of starches with tailored functional properties for food and non-food applications [148].

In addition, the development of yam-based snacks, such as fried or dehydrated chips, offers opportunities to generate higher-value and more market-attractive products [149,150]. While conventional frying conditions preserve desirable texture and color [151], growing consumer demand for healthier products highlights the need to explore alternative technologies, such as air frying, to reduce fat content without compromising sensory quality [152].

In the Colombian context, advancing yam valorization requires an integrated research agenda focused on postharvest standardization, optimization of processed products, and the full utilization of by-products within a circular bioeconomy framework [35,37,42]. These efforts are essential to consolidate yam as a strategic crop for agro-industrial diversification, food security, and sustainable rural development in the country’s main producing regions.

In parallel, the recovery and utilization of bioactive compounds such as diosgenin, phenolics, and flavonoids remain largely unexplored in Colombia and may represent an additional avenue for generating high-value functional ingredients from yam processing streams.

## 14. Research Priorities and Innovation Pathways for Yam-Based Products

Future research on yam flour should focus on improving its functionality and technological performance in bakery and gluten-free applications. Current evidence indicates that partial substitution of wheat flour can maintain acceptable quality and sensory characteristics, although higher substitution levels may adversely affect dough structure and bread quality [153,154]. Therefore, further studies on the modification of yam flour to enhance its properties and performance in bakery applications are of great importance.

Future research should also prioritize the optimization of starch extraction, purification, modification, and characterization methods to support industrial standardization and expanded applications [35,42,45]. Priority should therefore be given to large-scale processing, the development of physical, chemical, and enzymatic modification strategies, and a better understanding of starch interactions within complex food matrices [38,42,155].

From an environmental perspective, the partial replacement of maize starch with yam starch offers a sustainable alternative aligned with the principles of ecological production and the circular bioeconomy [35,37]. However, this potential requires confirmation through comprehensive life-cycle assessments and sustainability studies addressing energy use, land management, and waste valorization.

At the scientific level, several research priorities remain unresolved, including the standardization of starch characterization methods and nomenclature to facilitate comparison and reproducibility across studies, as well as the scaling-up of extraction and refinement processes with an industrial focus [42]. Genetic and biotechnological improvement aimed at developing varieties with either low or high amylose content (>40%) could further optimize functional properties [40,45]. Other relevant avenues include the evaluation of novel modification techniques, ultrasound, gamma irradiation, high pressure, or combined treatments, across a wider range of genotypes [38,146], as well as the exploration of structure–function–application relationships in different food matrices such as bakery, pasta, and biodegradable films [48].

Another important research priority concerns yam-derived bioactive compounds. *Dioscorea* spp. contain steroidal saponins (particularly diosgenin), phenolic compounds, and flavonoids that have demonstrated diverse biological activities in experimental studies [60,76]. Future research should prioritize the identification, quantification, bioaccessibility, and functionality of these compounds within realistic food matrices and dietary contexts.

In the Colombian context, further work is required to isolate and quantify these compounds, especially diosgenin and dioscin, in local cultivars from the Caribbean and Andean regions, and to determine their bioaccessibility and functional efficiency within food matrices through in vitro gastrointestinal digestion studies and biological assays assessing effects on oxidative-stress, inflammation, and glucose-metabolism markers [77,93]. Such research would not only expand the phytochemical knowledge of Colombian yam but also pave the way for the development of functional foods formulated with local bioactive ingredients, offering added value and market differentiation [78]. Future research should also explore enzyme-assisted extraction, biotransformation, and fermentation-based strategies to improve the recovery, bioaccessibility, and functionality of yam-derived bioactive compounds. These approaches may contribute to the sustainable development of value-added functional ingredients from *Dioscorea* spp.

Two cross-cutting gaps also deserve explicit attention. First, composition data across the yam literature are reported inconsistently on fresh- and dry-weight bases, which hampers direct comparison between studies, a limitation visible even within the present review. Second, there is no widely adopted, standardized protocol for quantifying diosgenin, which contributes substantially to the large variability reported in the literature.

Harmonization of reporting units, analytical methodologies, and compositional descriptors would significantly improve comparability, reproducibility, and data interpretation in future studies.

Addressing these research priorities will support the development of standardized yam-derived ingredients, facilitate industrial valorization, and strengthen the role of *Dioscorea* spp. within sustainable food systems and circular bio-economy strategies.

## 15. Conclusions

This review highlights yam as far more than a traditional subsistence crop, positioning it as a biologically complex, nutritionally relevant, and agro-industrially strategic resource. Across its diverse species, particularly *D. alata*, *D. rotundata*, and *D. cayenensis*, yam exhibits a combination of agronomic adaptability, compositional richness, and functional versatility that supports its importance for food security, rural livelihoods, and value-added applications in tropical and subtropical regions.

From a nutritional perspective, yam tubers provide a robust carbohydrate matrix dominated by starch, complemented by dietary fiber, minerals, vitamins, and proteins such as dioscorin. Beyond macronutrient supply, the phytochemical profile of yam, including steroidal saponins, polyphenols, flavonoids, tannins, alkaloids, and diosgenin, confers biological activities that extend its relevance to functional foods, nutraceuticals, and pharmaceutical precursors. Importantly, this review highlights the dual nature of several of these compounds, which may act as antinutritional factors in raw or poorly processed tubers but exert beneficial health effects when present at appropriate levels and consumed after adequate processing.

Processing emerges as a critical determinant of yam safety, quality, and functionality. Conventional domestic and industrial treatments, particularly boiling, soaking, and blanching, are highly effective in mitigating antinutritional factors while retaining or even enhancing bioactive potential. These findings reinforce that the nutritional and toxicological profile of yam is not an inherent limitation of the crop itself, but rather a function of preparation, processing, and cultivar selection.

At the agro-industrial level, yam flour, starch, and derived products represent underexploited opportunities for diversification and innovation. The functional properties of yam starch, together with the gluten-free nature of yam flour, open promising avenues for bakery, dairy, snack, and specialized food applications. However, variability among species, cultivars, and processing methods continues to limit standardization and large-scale adoption, highlighting the need for coordinated research on modification technologies, structure–function relationships, and processing optimization. Future progress will also require greater harmonization of analytical methodologies and reporting criteria, particularly regarding compositional data and the quantification of key bioactive compounds such as diosgenin.

In the Colombian context, yam valorization holds strategic relevance. Despite its socioeconomic importance, production systems remain constrained by limited technological input, post-harvest losses, genetic erosion, and restricted industrial integration. Strengthening germplasm conservation, improving planting material quality, and integrating biotechnological, post-harvest, and processing innovations are essential steps toward consolidating yam as a driver of sustainable rural development and circular bioeconomy initiatives.

Overall, this review emphasizes that yam occupies a unique intersection between staple nutrition and high-value functionality. Advancing its full potential will require interdisciplinary approaches that integrate agronomy, food science, biotechnology, and socio-economic research. Such efforts will not only enhance the competitiveness of yam-based systems but also reinforce the role of *Dioscorea* spp. as resilient, multifunctional crops in future agri-food systems.

## Figures and Tables

**Table 1:** Comparative description of the main morphological and productive characteristics of three *Dioscorea* spp. present in agricultural systems of the Colombian Caribbean.

Morphological Characteristic	*D. alata*	*D. rotundata*	*D. cayenensis*	References
Presence of spines	Generally absent	Present	Present	[23]
Twining direction	Variable; used as a diagnostic character	Variable	Variable	[23]
Tuber shape	Cylindrical to oval	Oval to cylindrical	Oval to cylindrical	[18,19]
Tuber flesh color	White	White	Yellow	[19]
Number of tubers per plant	Variable (trait used for species identification)	Variable	Variable	[23]
Morphological differentiation among species	High intraspecific variability	Distinguishable from *D. cayenensis*	Distinguishable from *D. rotundata*	[24]
Yield (t ha^−1^)	10–25	4–9	4–9	[25]
Production cycle	Annual	Annual	Annual	[25]

**Table 2:** Representative proximate composition (g/100 g FW) of *D. alata*, *D. rotundata* and *D. cayenensis*.

Yam Species	Moisture	Crude Protein	Crude Fat	Crude Fibre	Ash	Starch
*D. alata*	64.9–87.8 ^a^	0.9–2.1 ^b^	0.03–0.3 ^b^	0.4–0.6 ^b^	0.6–2.2 ^b^	13.2–19.7 ^b^
*D. rotundata*	54.5–75.2 ^a^	1.2–1.8 ^b^	0.05–0.2 ^b^	0.4–1.9 ^b^	0.8–0.9 ^b^	21.1–26.2 ^b^
*D. cayenensis*	62.2–89.4 ^a^	1.7 ^c^	0.16 ^c^	0.7 ^c^	1.7 ^c^	ND ^d^

Proximate composition values are expressed on a FW basis (g/100 g FW). Values originally reported on a dry weight (DW) basis were converted to FW using the moisture contents reported for the same samples in the original studies according to the equation: FW = DW × (100 − moisture)/100; Variability in the reported results reflects interspecific and genotypic differences, environmental conditions, and methodological variation among analytical approaches. ^a^ Range values from [6]; ^b^ Range values from [7,8]; ^c^ Average values derived from [8]; ^d^ ND (not determined), no analytically determined starch value was identified in the selected reference. Polycarp et al. (2012) [8] reported carbohydrates by difference, which were converted to an estimated fresh-weight value of 25.1 g/100 g FW but were not considered directly comparable with analytically determined starch values and therefore were not included in the table.

**Table 3:** Average mineral composition (mg/100 g) of *D. alata*, *D. rotundata* and *D. cayenensis*.

Yam Species	K	Na	Ca	Mg	P	Fe	Cu	Mn	Zn
*D. alata*	5.2–3933 ^a^	0.3–380 ^a^	0.22–3032 ^a^	0.6–656.3 ^a^	26.6–340 ^a^	0.7–124.3 ^a^	0.1–11.2 ^b^	0.5–6.4 ^a^	0.4–6.8 ^b^
*D. rotundata*	475–1591	10.4–87.5 ^b^	22.8–114.4 ^a^	35.5–53 ^c^	27–211.5 ^b^	5–78.3 ^b^	0.2 ^d^	1.1–1.8 ^c^	0.35–6.8 ^b^
*D. cayenensis*	700–825 ^c^	62.5–70 ^c^	74.5–82 ^c^	38–57.5 ^c^	19.15–190.5 ^b^	5–27.9 ^b^	0.2 ^d^	1.2 ^d^	0.7–5.85 ^b^

Mineral composition values are expressed on a DW basis (mg/100 g DW); Variability in the reported results reflects interspecific and genotypic differences, environmental conditions, and methodological variation among analytical approaches. ^a^ Range values from [6]; ^b^ Range values from [6,8]; ^c^ Average values derived from [8]; ^d^ Average values derived from [8]. The wide variability observed for some minerals reflects differences among studies in yam genotype, sample type, environmental conditions, analytical methodologies, and reporting basis (fresh-weight or dry-weight), in addition to biological variation. Therefore, direct comparisons among studies should be interpreted with caution.

**Table 4:** Average concentrations of selected major antinutritional factors (mg/100 g) in *D. alata*, *D. rotundata*, and *D. cayenensis*.

Yam Species	Oxalates	Phytates	Tannins	Alkaloids	Saponins
*D. alata*	0.4–1.2 ^a^	3–3.1 ^c^	10.7–13.2 ^c^	NR	2.2–3.8 ^e^
*D. rotundata*	0.6–3.2 ^b^	2.5–2.6 ^c^	4.6–62 ^b^	11.6 ^d^	NR
*D. cayenensis*	0.5–4.9 ^b^	3.2–4.2 ^c^	4.4–30.1 ^b^	8.3 ^d^	NR

Values are reported as presented in the original references. The original studies used different analytical methodologies and, in some cases, reported results on different bases (FW or DW) or did not explicitly specify the reporting basis; therefore, these values should be interpreted as indicative literature ranges rather than directly comparable quantitative measurements; variability in the reported results reflects interspecific and genotypic differences, environmental conditions, and methodological variation among analytical approaches. ^a^ Range values derived from [8,72]. ^b^ Range values derived from [8,68]). ^c^ Range values from [8]. ^d^ Average values from [68]. ^e^ Range values from [73]. NR = Not reported.

**Table 5:** Principal bioactive compounds reported in yam.

Compound	Description & Occurrence	Evidence/Typical Concentrations	Functions/Biological Relevance	References
Diosgenin	A steroidal sapogenin widely reported in several *Dioscorea species* (e.g., *D. zingiberensis*, *D. alata*, *D. deltoidea*). Biosynthetic and genetic determinants have been identified in some species.	Concentrations are species- and genotype-dependent; genome wide association study approach (GWAS) studies identify loci controlling diosgenin in *D. zingiberensis*.	Precursor for steroidal drug synthesis; investigated in experimental models for anti-inflammatory, cholesterol-modulating, and antimicrobial activities.	[73,82,83]
Steroidal saponins (incl. other saponins)	Glycosylated steroidal compounds abundant in many *Dioscorea* spp.; structural diversity includes furostanol and spirostanol types.	Detected in multiple *Dioscorea* species; reported concentrations vary according to species, cultivar, harvest stage, and analytical methodology;. New steroid saponins described in *D. zingiberensis* with demonstrated bioactivity.	Structurally diverse metabolites associated with anti-inflammatory, antioxidant, and cholesterol-modulating activities in experimental studies; important precursors of diosgenin; promising for TRL ≥ 4 applications in food/pharma delivery discussed in reviews.	[77,84,85]
Flavonoids	Polyphenolic compounds (flavones, flavonols, etc.) found in flesh and peels of various *Dioscorea* species.	Reported in nutritional and phytochemical surveys.	Contribute to antioxidant capacity and may influence oxidative stability and functional properties of yam-derived products.	[6,78,86]
Polyphenols (non-flavonoid phenolics)	Includes phenolic acids and related compounds; contribute to antioxidant properties.	Quantified in compositional studies and functional-food reviews; levels vary with species and processing.	Associated with antioxidant capacity and interactions with food matrices that may influence bioaccessibility and functionality; often co-occurs with flavonoids and tannins.	[6,58]
Alkaloids	Nitrogen-containing secondary metabolites reported in some *Dioscorea* spp.; occurrence is species-specific and often minor compared to saponins.	Mentioned in genus-wide reviews and ethnopharmacological surveys.	May contribute to biological activity but are also relevant for safety assessment because some alkaloids are associated with bitterness and toxicity in wild species.	[76,87]
Tannins	High-molecular-weight polyphenolics present in some yam species; associated with astringency and potential antinutritional effects.	Measured in compositional studies and processing-oriented safety work (e.g., processing effects on flour/chips).	Processing often reduces tannin levels; relevant for digestibility and sensory attributes.	[8,75]
Dioscorin	Major tuber storage protein in many *Dioscorea* spp.; multifunctional (antioxidant, protease inhibitor activities reported).	Reported as the principal tuber storage protein; content varies by species and processing.	Important for nutritional quality and functional properties in food processing; potential bioactivities beyond nutrition.	[6,88,89,90]

**Table 6:** Patents involving yam and its bioactive compounds in cosmetic, pharmaceutical, and food applications.

Patent	Technical Summary	Applications	Reference
EP-3167074-A1	Reports compositions based on *Dioscorea* spp. extract or diosgenin derivatives for topical or ingestible formulations.	Cosmetic: skin regeneration, antioxidant Pharmaceutical: anti-inflammatory Food: nutraceutical ingredient	[131]
WO-2019142760-A1	Discloses use of *D. alata* or related species in bioactive formulations.	Cosmetic: anti-aging Pharmaceutical: metabolic regulation Food: functional foods	[132]
US-2019060341-A1	Describes compositions including extracts from *D. oppositifolia* or *D. villosa* rich in diosgenin.	Cosmetic: UV protection and moisturizing Pharmaceutical: antidiabetic and hormone-regulating uses Food: dietary supplements	[133]
IT-202000007285-A1	Describes technological process using *D. zingiberensis* saponins for functional and medicinal preparations.	Cosmetic: bioactive emulsions Pharmaceutical: anti-inflammatory and cholesterol-regulating drugs	[134]
JP-H1067670-A	Related to formulations or processes using *D. japonica* or *D. oppositifolia* for therapeutic or beauty-related products.	Cosmetic: whitening, anti-wrinkle Pharmaceutical: hormonal balance Food: fortified functional products	[135]

## Data Availability

The original contributions presented in the study are included in the article, further inquiries can be directed to the corresponding author.
